# The role of contrast-enhanced transcranial color-coded real-time sonography combined with the common carotid artery compression test in preoperative evaluation of anterior communicating artery patency in patients with insufficient temporal bone windows

**DOI:** 10.3389/fneur.2026.1806901

**Published:** 2026-09-01

**Authors:** Ying Liu, Shuai Zheng, Yuyang Gan, Sen Wang, Wen He, Wei Zhang

**Affiliations:** Department of Ultrasound, Beijing Tiantan Hospital, Capital Medical University, Beijing, China

**Keywords:** anterior communicating artery, carotid endarterectomy, common carotid artery compression test, contrast-enhanced transcranial color-coded real-time sonography, symptomatic carotid artery stenosis

## Abstract

**Objective:**

The purpose of the study was to evaluate the anterior communicating artery (ACoA) patency in patients with suboptimal temporal bone windows who were scheduled for carotid endarterectomy (CEA), using contrast-enhanced transcranial color-coded real-time sonography (CE-TCCS) combined with common carotid artery compression (CCC) test.

**Method:**

A prospective analysis was conducted on 116 patients with suboptimal temporal bone windows who exhibited symptomatic carotid artery stenosis (sCAS). The display rates of intracranial circle of Willis (CoW) including anterior cerebral artery (ACA), middle cerebral artery, and posterior cerebral artery, were analyzed based on TCCS and CE-TCCS data. The functional patency of the ACoA was assessed by CE-TCCS with and without the CCC test, and the results were compared with the anatomical patency determined by CTA.

**Result:**

The visualization rates of CoW were significantly improved in CE-TCCS (*p* < 0.001). CE-TCCS excluded 24 patients due to unilateral absence of the ACA-A1 segment. We assessed the patency of the ACoA in 92 patients. With CTA as the anatomical reference standard, CE-TCCS combined with CCC test yielded 94.67% sensitivity, 100% specificity and positive predictive value, 80.95% negative predictive value, and 95.65% overall diagnostic accuracy. Compared with CTA, CE-TCCS combined with CCC test showed no significant difference (*p* = 0.125) and excellent consistency (Kappa = 0.868, 95% CI: 0.743–0.993, *p* < 0.001), whereas standalone CE-TCCS differed significantly from CTA (*p* < 0.001) with poor consistency (Kappa = 0.126, 95% CI: 0.053–0.199, *p* = 0.013). The functional ACoA patency rate detected by CE-TCCS combined with CCC test was 77.2%, while 70.4% of patent cases were missed by CE-TCCS alone.

**Conclusion:**

CE-TCCS significantly improve the display rate of intracranial CoW. CE-TCCS combined with the CCC test can serve as a reliable technique in preoperative evaluation of ACoA patency in patients with inadequate temporal bone windows.

## 1 Introduction

Approximately 15% to 20% of ischemic strokes result from large artery atherosclerosis, with a significant proportion specifically linked to extracranial carotid artery stenosis (CAS) (1, 2). Although CEA does not lead to a significant benefit for asymptomatic CAS (3), it remains the recommended treatment especially for sCAS and is regarded as an effective strategy for preventing stroke (4). However, carotid clamping during CEA can lead to intraoperative ischemia and potentially increase the risk of neurological complications. To mitigate this risk, surgeons must determine whether to use a temporary arterio-arterial shunt or perform the procedure without one. Although shunting offers potential benefits, its use is associated with an increased risk of perioperative stroke or mortality (5). Possible complications include air embolism, plaque embolism, arterial dissection, or acute arterial occlusion. Moreover, the use of a shunt prolongs the duration of the surgical procedure (6). Placement of a shunt should be considered at the discretion of the operating surgeon (7). Many surgeons shunt selectively if the CoW is incomplete (5). Previous study has demonstrated that the absence of an ACoA has been associated with a higher incidence of new ischemic lesions following CEA (8). As the key pathway of collateral flow, the patency of the ACoA can serve as an important predictive indicator for determining the necessity of shunt placement during the procedure (9–11).

In 1911, Matas (12) described temporary arterial occlusion by manual compression of the common carotid artery to test the competence of the collateral circulation. Padayachee et al. (13) first reported in 1986 that transcranial Doppler sonography (TCD) combined with the CCC test allows collateral functional assessment. Nevertheless, TCD cannot directly display vascular structures, and angle-corrected flow velocity measurements are unfeasible, potentially reducing diagnostic accuracy. Transcranial color-coded real-time sonography (TCCS) is now widely used instead of TCD. This specialized color-coded imaging modality integrates two-dimensional grayscale, color Doppler, and spectral Doppler imaging to clearly visualize intracranial vasculature (14). However, limited acoustic penetration via the temporal bone window results in markedly suboptimal visualization of the CoW among elderly patients. In 1988, Postert et al. (15) used galactose-based microbubbles to produce accurate contrast in different brain regions. With the advent of novel microbubble contrast agents capable of pulmonary transit, CEUS involves the use of 1–5-μm lipid-shelled microbubbles as purely intravascular agents to visualize intracranial vascular blood flow in real time (16). When insonated, microbubbles agents oscillate nonlinearly and produce strong contrast signals because the gas core is acoustically mismatched with surrounding tissues (17). Previous studies have shown that use of intravenously administered contrast material can almost completely overcome the limitations associated with poor temporal acoustic windows (16, 18).

Building on previous research, we adopted CE-TCCS combined with the CCC test as the research method to assess the functional patency of the ACoA, and CTA was used as the imaging modality to assess the anatomical patency. We explored whether functional patency of the ACoA corresponds to its anatomical patency shown on CTA. Our objective was to establish a reliable protocol for preoperative evaluation of ACoA patency in patients with inadequate temporal bone windows prior to CEA.

## 2 Methods

### 2.1 Patients

**Figure 1:**
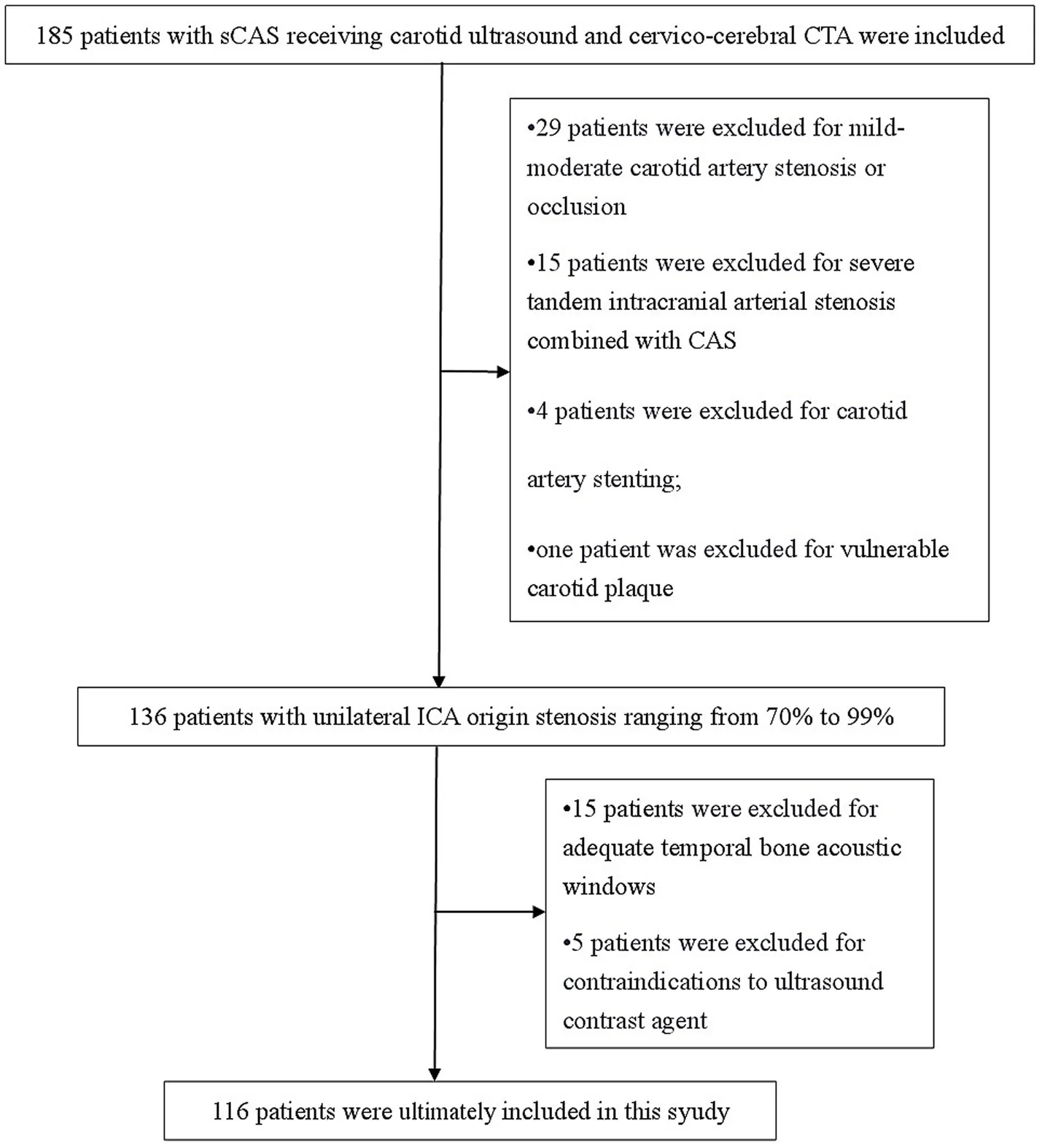
Flowchart of the study.

A total of 185 patients were initially screened between March 2024 and December 2025. Ultimately, 116 patients with ≥70% severe stenosis at the origin of the internal carotid artery (ICA) and insufficient temporal acoustic bone window were enrolled in the study. A study flowchart is presented in Figure 1. All patients had experienced an ipsilateral transient ischemic attack (TIA) or ischemic stroke within 6 months and were planned for CEA. The severity of ICA stenosis was determined using carotid Doppler ultrasonography (19), and confirmed by neck CTA. The hemisphere ipsilateral to the sCAS scheduled for CEA was defined as the CEA side, and the opposite hemisphere as the contralateral side. Clinical data was shown in Table 1.

**Table 1:** Clinical data collection.

Clinical data	Value
Severity of ICA stenosis on CEA side	
70–99%, *n* (%)	116 (100)
Severity of ICA stenosis on contralateral side	
<50%, *n* (%)	93 (80.2)
50–69%, *n* (%)	23 (19.8)
70–99% or occlusion, *n* (%)	0 (0)
Age(yr) (mean)	64 ± 7
Male sex, *n* (%)	100 (86.2)
BMI (kg/m^2^)	27.9 ± 3.6
SBP (mm/Hg)	134 ± 11.5
DBP (mm/Hg)	84.2 ± 9.3
Smoking, *n* (%)	46 (39.7)
Drinking, *n* (%)	34 (29.3)
Symptoms	
Limb weakness, numbness, or limb motor dysfunction, *n* (%)	67 (57.8)
Speech difficulties and facial asymmetry, *n* (%)	31 (26.7)
Ocular symptoms, *n* (%)	23 (19.8)
TIA, *n* (%)	79 (68.1)
Infarct on CT/MRI, *n* (%)	37 (31.9)
Comorbidities	
Hypertension, *n* (%)	65 (56.0)
Mellitus, *n* (%)	26 (22.4)
Hyperlipidemia, *n* (%)	19 (16.4)
Medication	
Lipid-lowering agents, *n* (%)	69 (59.5)
Antihypertensive agents, *n* (%)	61 (52.3)
Hypoglycemic agents, *n* (%)	24 (20.7)

### 2.2 Inclusion criteria

(i) Age between 18 and 80 years;
(ii) 70%–99% stenosis of the ICA origin with sCAS;
(iii) Patients with insufficient temporal bone acoustic window;
(iv) Availability of complete neck and head ultrasonography data, CTA images, and clinical records, with an interval of less than 3 months between ultrasonography and CTA.

### 2.3 Exclusion criteria

(i) Asymptomatic carotid artery stenosis;
(ii) Mild–moderate carotid artery stenosis or occlusion;
(iii) Non-atherosclerotic carotid artery stenosis;
(iv) Presence of contraindications to ultrasound contrast agent or iodine contrast agent;
(v) Severe tandem intracranial arterial stenosis combined with CAS;
(vi) Carotid artery stenting;
(vii) Vulnerable carotid plaques.

### 2.4 Methods

#### 2.4.1 CE-TCCS

The CE-TCCS examinations were performed by investigators with more than 5 years of experience, who were blinded to the CTA results. TCCS was implemented using a PHILIPS EPIQ 7 color Doppler ultrasound instrument, equipped with a phased array probe of a practical frequency of 1.0–5.0 MHz. For conventional TCCS examination, the patient was placed in the supine position. The bilateral A1 and A2 segments of the ACA, M1 and M2 segments of the middle cerebral artery (MCA), and P1 and P2 segments of the posterior cerebral artery (PCA) were scanned through the temporal window, and any unsatisfactory visualization was documented. An insufficient temporal bone window was defined as the inability to adequately visualize flow signals from any segment of the ACA, MCA, or PCA. Before the contrast agent SonoVue (Bracco, Milan, Italy) was utilized, the freeze-dried powder (59 mg) under a sulfur hexafluoride (SF6) atmosphere was reconstituted with 5 mL of normal saline; a 0.5-mL dose was then injected into the antecubital vein via bolus injection, followed by a flush of 5 mL of normal saline after each administration (18). Through the temporal window, the mechanical index, color gain, and wall filter were adjusted to optimal settings under the color Doppler imaging mode to avoid the “blooming artifact “resulting from the excessively high concentration of the contrast agent in the early stage. The segments of the CoW, including the ACA, MCA, and PCA, were required to be clearly visualized. A vascular segment was considered visualized when continuous opacification of the target vessel for 2–6 min was observed on CE-TCCS images. The integrity, contrast filling status, flow direction, and hemodynamic parameters of each segment were documented. Consistent with color Doppler conventions, flow toward the probe was displayed in red, while flow away from the probe was displayed in blue.

#### 2.4.2 CCC test

Before CCC test, a carotid artery color Doppler ultrasound examination should be performed to prevent the shedding of vulnerable plaques. The carotid artery ultrasound implemented using a PHILIPS EPIQ 7 color Doppler ultrasound instrument, equipped with a linear array probe of a practical frequency of 3.0–12.0 MHz. The position for compressing the CCA should be at the proximal segment of the CCA at the level of the supraclavicular fossa, not at the level of the thyroid cartilage or above. The patient should be placed in a supine position, and static compression should be applied for no more than four cardiac cycles per session. The technique should be gentle and should not be repeated multiple times (11, 20).

#### 2.4.3 CE-TCCS combined with CCC test for assessing whether the ACoA was functionally patent

##### 2.4.3.1 Patency of the ACoA

(1) Blood flow signals from both ACA-A1 segment are normal displayed, with the blood flow directed away from the probe. When the CCA is compressed on CEA side, the blood flow velocity of the ipsilateral ACA-A1 segment decreases, and a reverse compensatory blood flow signal appears; while the blood flow velocity of the contralateral ACA-A1 segment increases. (2) In some cases with severe stenosis on CEA side, the blood flow in the ACA-A1 segment on the CEA side reverses, directing the flow toward the prob. When the CCA is compressed on the contralateral side, the blood flow velocity in the contralateral ACA-A1 decreases, and the blood flow velocity in the ACA-A1 segment also decreases on the CEA side (21).

##### 2.4.3.2 No patency of the ACoA

Blood flow signals from both ACA-A1 segment are normally displayed, directing the flow away from the probe. When the CCA is compressed on CEA side, the blood flow velocity in the ipsilateral ACA-A1 segment is markedly reduced or even absent, with no evidence of reverse compensatory flow, whereas the flow velocity in the contralateral ACA-A1 segment remains unchanged (21).

All variations in flow direction and flow velocity values following carotid compression were recorded.

#### 2.4.4 CE-TCCS for assessing whether the ACoA was functionally patent

##### 2.4.4.1 Patency of the ACoA

The blood flow in the ACA-A1 segment on the CEA side reverses, directing the flow toward the probe (22).

##### 2.4.4.2 No patency of the ACoA

Blood flow signals from both ACA-A1 segment are normal displayed, directing the flow away from the probe (22).

#### 2.4.5 CTA for assessing anatomical patency of the ACoA

Head and neck CTA was performed on a Revolution CT Scanner (GE Healthcare, USA) using 256-slice helical scanning mode with high-resolution imaging capabilities. Scanning direction was caudocranial. 65 mL nonionic iodinated contrast medium (Omnipaque, Shanghai, China; 350 mg I/mL) was administered via the antecubital vein at an injection rate of 4 mL/s with a high-pressure syringe, followed by a 40 mL saline flush injected at 4 mL/s. Images were acquired with the following technical parameters: section thickness, 0.625 mm; collimation, 0.625 mm; gantry rotation speed, 600 ms; 120 kVp; 450 mAs; FOV, 200–250 mm; and 512 × 512 matrix. The obtained images were sent to the workstation via local network. 3D-MIP reconstruction and 3D-VR reconstruction were performed. Image interpretation was performed by two experienced neuroradiologists, with at least 5 years of experience. A 5-point grading scale proposed by Sundaram et al. (23) was adopted to evaluate the anatomical patency of the ACoA: grade 1, absent; grade 2, probably present; grade 3, hairline; grade 4, definitely present; grade 5, robust. Grades 3–5 were classified as patent ACoA, whereas Grades 1–2 were defined as non-patent ACoA.

### 2.5 Statistical analysis

SPSS 26.0 statistical software was used for data analysis. Normally distributed measurement data were expressed as mean ± standard deviation, while counting data were presented as *n* or %. McNemar’s test was used to compare the visualization rates of ACA A1/A2 segments, MCA M1/M2 segments, and PCA P1/P2 segments between conventional TCCS and CE-TCCS. Bonferroni correction was applied, and the adjusted significance level was set to approximately 0.0083. Sensitivity, specificity, positive, negative predictive values, and accuracy of CE-TCCS combined with CCC were calculated with CTA as the reference standard. The 95% confidence intervals were calculated using the Clopper-Pearson exact method. McNemar’s test along with the Cohen’s Kappa consistency test was utilized to assess differences and consistency between the two inspection methods. The significance level was defined as *p* < 0.05.

## 3 Results

### 3.1 Comparison of the display rate of the intracranial CoW between conventional TCCS and CE-TCCS

**Figure 2:**
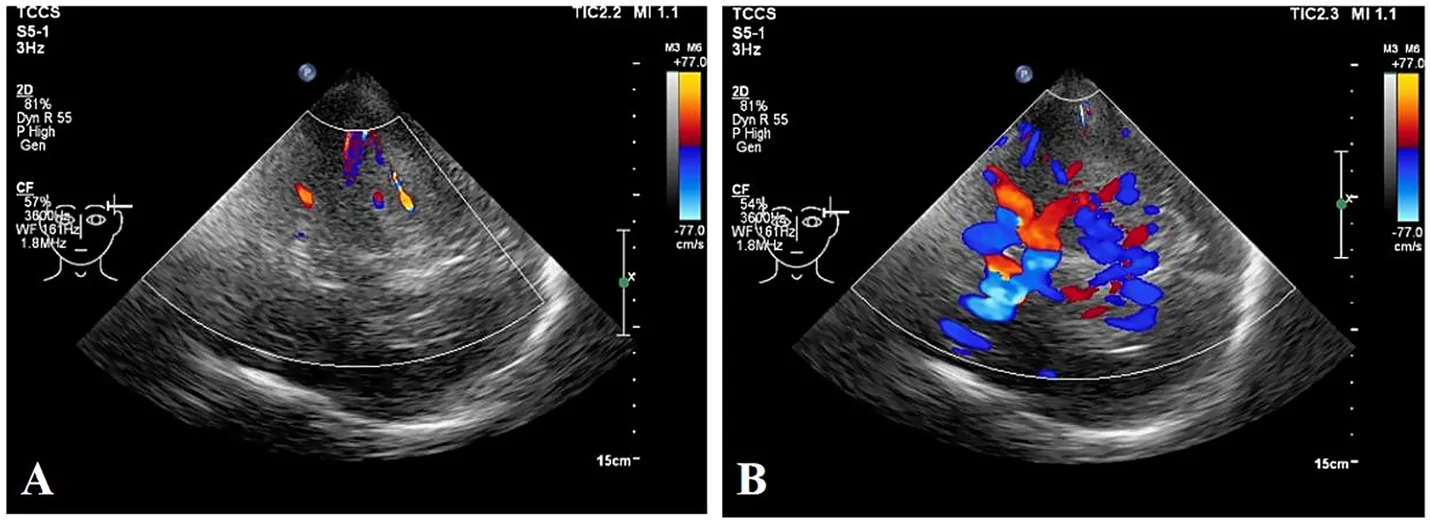
**(A)** Conventional TCCS was unable to visualize any intracranial blood vessel due to the poor temporal acoustic window on the left. **(B)** The CE-TCCS showed blood flow signals of the CoW, including bilateral ACAs, MCAs, and PCAs.

**Figure 3:**
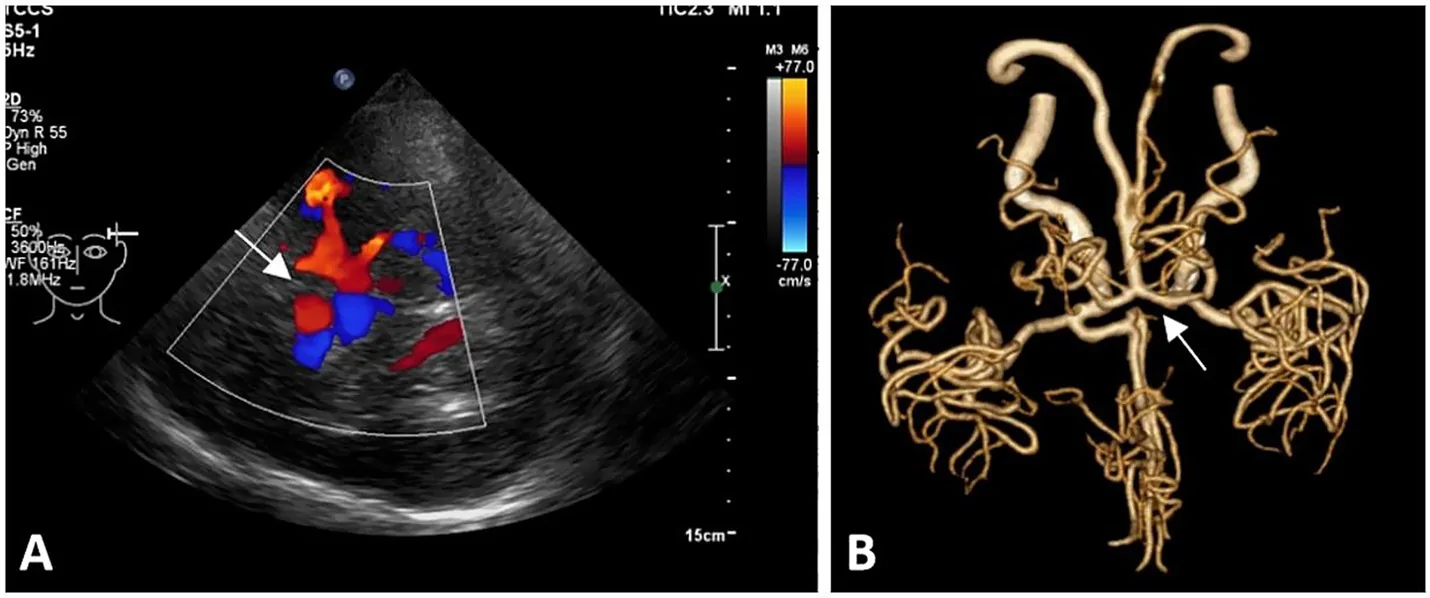
**(A)** Lack of blood flow signal in the left ACA-A1 segment, as observed by CE-TCCS through the left temporal window (white arrow). **(B)** CTA confirmed the absence of the left ACA A1 segment (white arrow).

**Figure 4:**
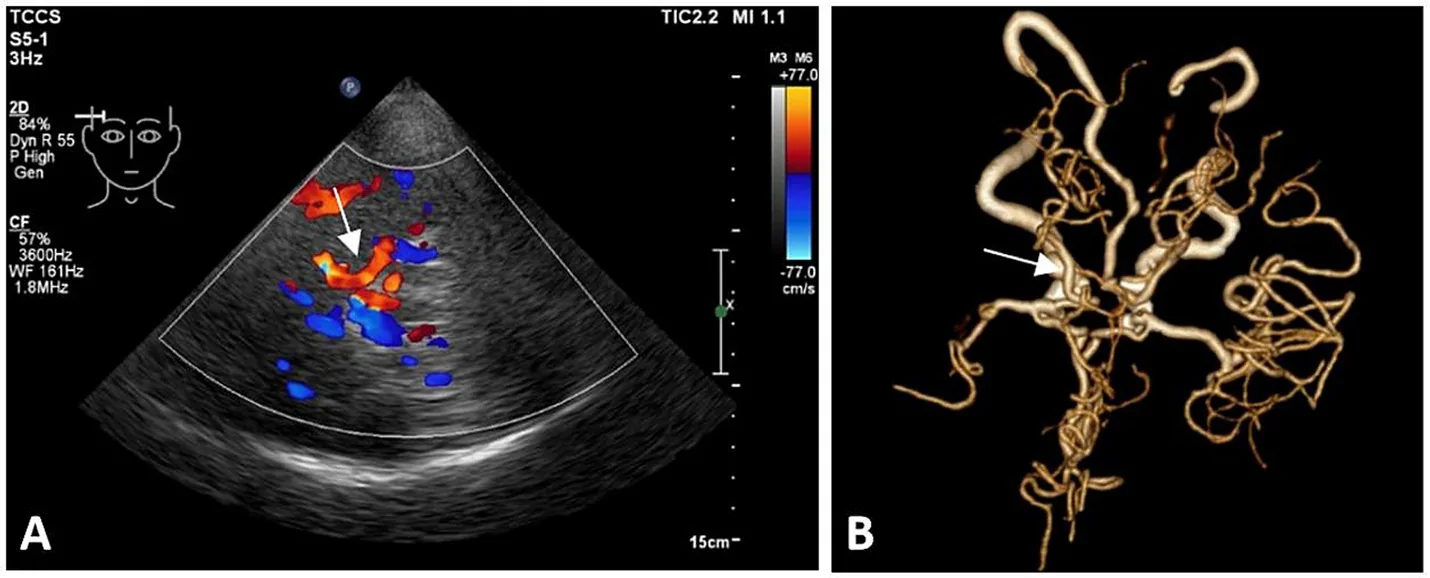
**(A)** CE-TCCS showed that the right p1 segment of the PCA was not visualized, and the p2 segment was entirely supplied by the ipsilateral ICA via the posterior communicating artery (white arrow). **(B)** CTA confirmed the presence of a right complete embryonic-type PCA (white arrow).

The visualization of ACA, MCA, and PCA were significantly improved in CE-TCCS compared with TCCS (Figure 2). Table 2 reported the display rates of conventional TCCS and CE-TCCS for bilateral ACA, MCA, and PCA segments in 116 patients (*p* < 0.001) 24 patients with non-visualized unilateral ACA-A1 segments on CE-TCCS, and CTA confirmed congenital absence of these ACA-A1 segments (Figure 3). Fourteen cases with non-visualized unilateral PCA-P1 segments on CE-TCCS; the entire P2 segment was supplied by the ipsilateral ICA through the posterior communicating artery, consistent with unilateral complete embryonic-type PCA verified by CTA (Figure 4).

**Table 2:** Display rate of the intracranial CoW by TCCS and CE-TCCS (*n* = 116).

Vascular segment	TCCS (%)	CE-TCCS (%)	*p*
ACA A1	38.4 (89/232)	89.7 (208/232)	<0.001
A1	48.7 (113/232)	100.0 (232/232)	<0.001
MCA M1	65.5 (152/232)	100.0 (232/232)	<0.001
M1	60.3 (140/232)	99.1 (230/232)	<0.001
PCA P1	32.8 (76/232)	94.0 (218/232)	<0.001
P1	49.6 (115/232)	100.0 (232/232)	<0.001

### 3.2 Comparison of the anatomical and functional patency of the ACoA

CE-TCCS excluded 24 patients due to unilateral congenital absence of the ACA-A1 segment. With CTA as the anatomical reference standard, we assessed the functional patency of the ACoA in 92 patients using both the CE-TCCS combined with CCC test and the independent CE-TCCS. Tables 3, 4 compared CE-TCCS combined with CCC and CE-TCCS alone against CTA for ACoA patency.

**Table 3:** Diagnostic results of CE-TCCS combined with CCC test and CTA for the patency of ACoA (*n* = 92).

Method	Result	CTA		Total
		Patent	Non-patent (absent)	
CE-TCCS combined with CCC Test	Patent	71	0	71
	Non-patent	4	17	21
Total		75	17	92

**Table 4:** Diagnostic results of CE-TCCS and CTA for the patency of ACoA (*n* = 92).

Method	Result	CTA		Total
		Patent	Non-patent (absent)	
CE-TCCS	Patent	21	0	21
	Non-patent	54	17	71
Total		75	17	92

#### 3.2.1 CE-TCCS combined with CCC test and CTA evaluating ACoA patency

Compared with CTA, the diagnostic performance of combined CE-TCCS and CCC was as follows: sensitivity of 94.67% (95% CI: 86.92%–98.20%), specificity of 100.00% (95% CI: 80.41%–100.00%), positive predictive value of 100.00% (95% CI: 94.94%–100.00%), negative predictive value of 80.95% (95% CI: 58.09%–94.55%), and overall accuracy of 95.65% (95% CI: 88.04%–98.80%). No statistically significant difference was observed (*p* = 0.125), and a high level of consistency was demonstrated (Kappa = 0.868, 95% CI: 0.743–0.993, *p* < 0.001).

**Figure 5:**
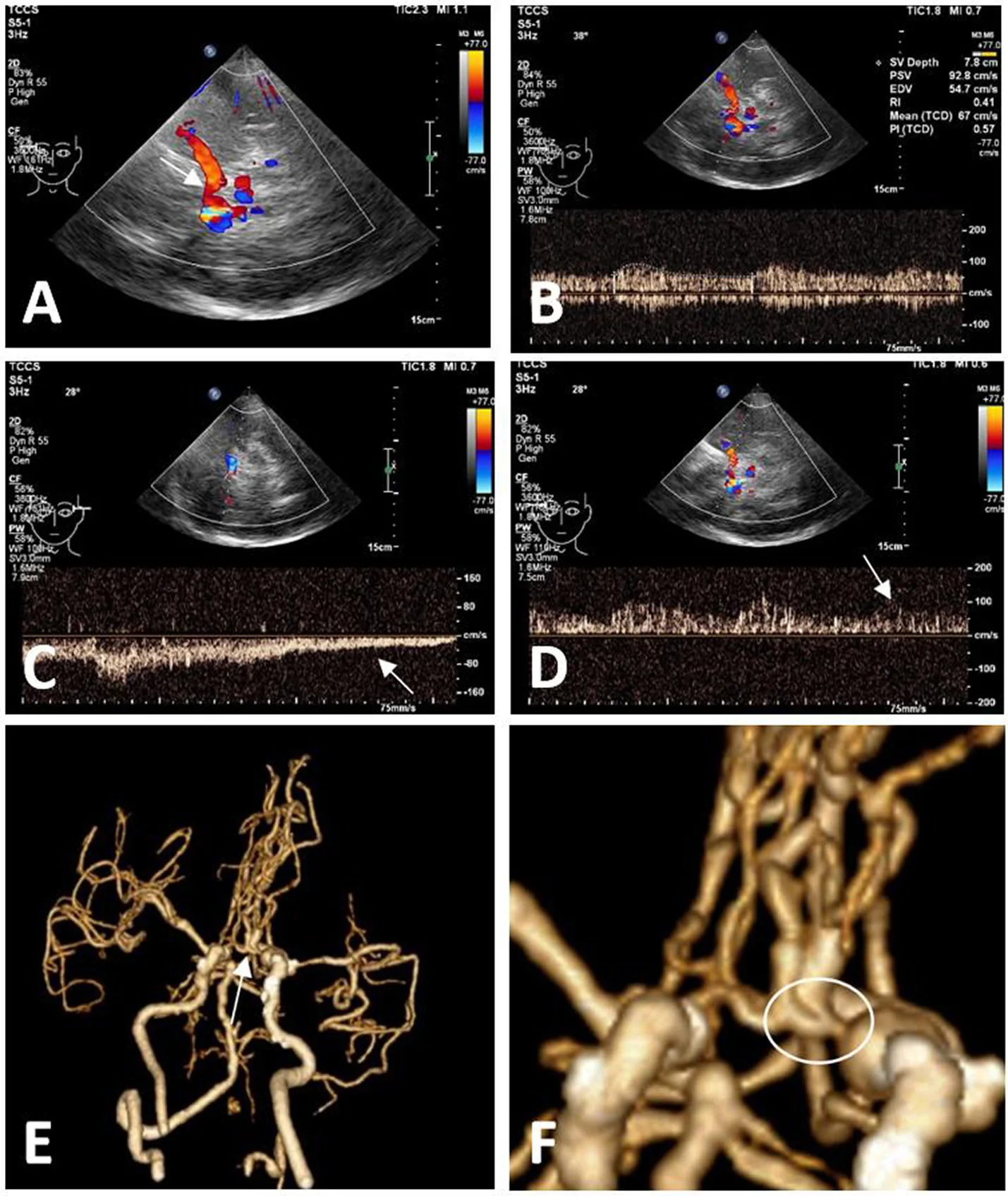
A male patient with 70%–99% stenosis at the origin of the right ICA, where the right side corresponded to the side of CEA. **(A,B)** The blood flow in the ACA-A1 segment on the CEA side reversed at rest, directing the flow toward the probe and PSV 92 cm/s (white arrow). **(C,D)** When the CCA was compressed on the contralateral side, the blood flow velocity of the contralateral ACA-A1 segment decreased (white arrow), and the blood flow velocity of the ACA-A1 segment also decreased on the CEA side (white arrow). **(E,F)** CTA confirmed the patency of the ACoA.

**Figure 6:**
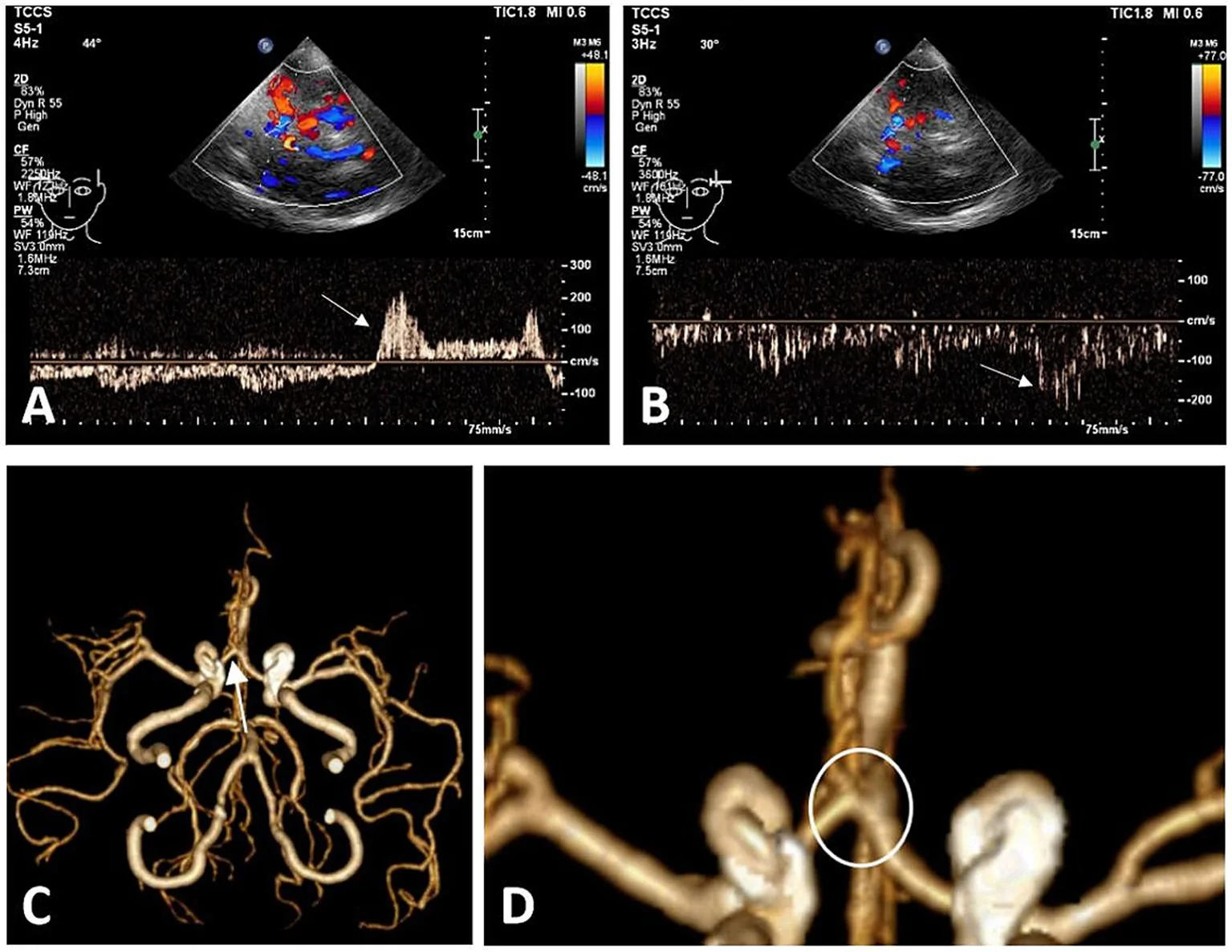
A male patient with 70%–99% stenosis at the origin of the right ICA, where the right side corresponded to the side of CEA. **(A,B)** Blood flow signal from both ACA -A1 segment was normal at rest, directing the flow away from the probe. When the CCA was compressed on CEA side, the blood flow velocity of the ipsilateral ACA-A1segment decreased, and a reverse compensatory blood flow signal appeared (white arrow), while the blood flow velocity of the contralateral ACA-A1 segment increased (white arrow). **(C,D)** CTA confirmed the patency of the ACoA.

CE-TCCS combined with the CCC test identified 71 cases with functionally patent ACoA, these results were consistent with CTA findings, yielding a patency rate of 77.2% (71/92). Among the 71 patients with patent ACoA, 29.6% (21/71) presented reversed flow in the ACA-A1 segment on the CEA side at rest. During contralateral CCA compression, the flow velocity of the contralateral ACA-A1 segment decreased, accompanied by a reduction in flow velocity of the ipsilateral CEA-side ACA-A1 segment (Figure 5). While 70.4% (50/71) of patients with patent ACoA showed normal flow in the ACA-A1 segment on the CEA side at rest, CCA compression at the CEA side induced compensatory reversed flow in the ipsilateral ACA-A1 segment, and flow velocity in the contralateral ACA-A1 segment increased (Figure 6).

**Figure 7:**
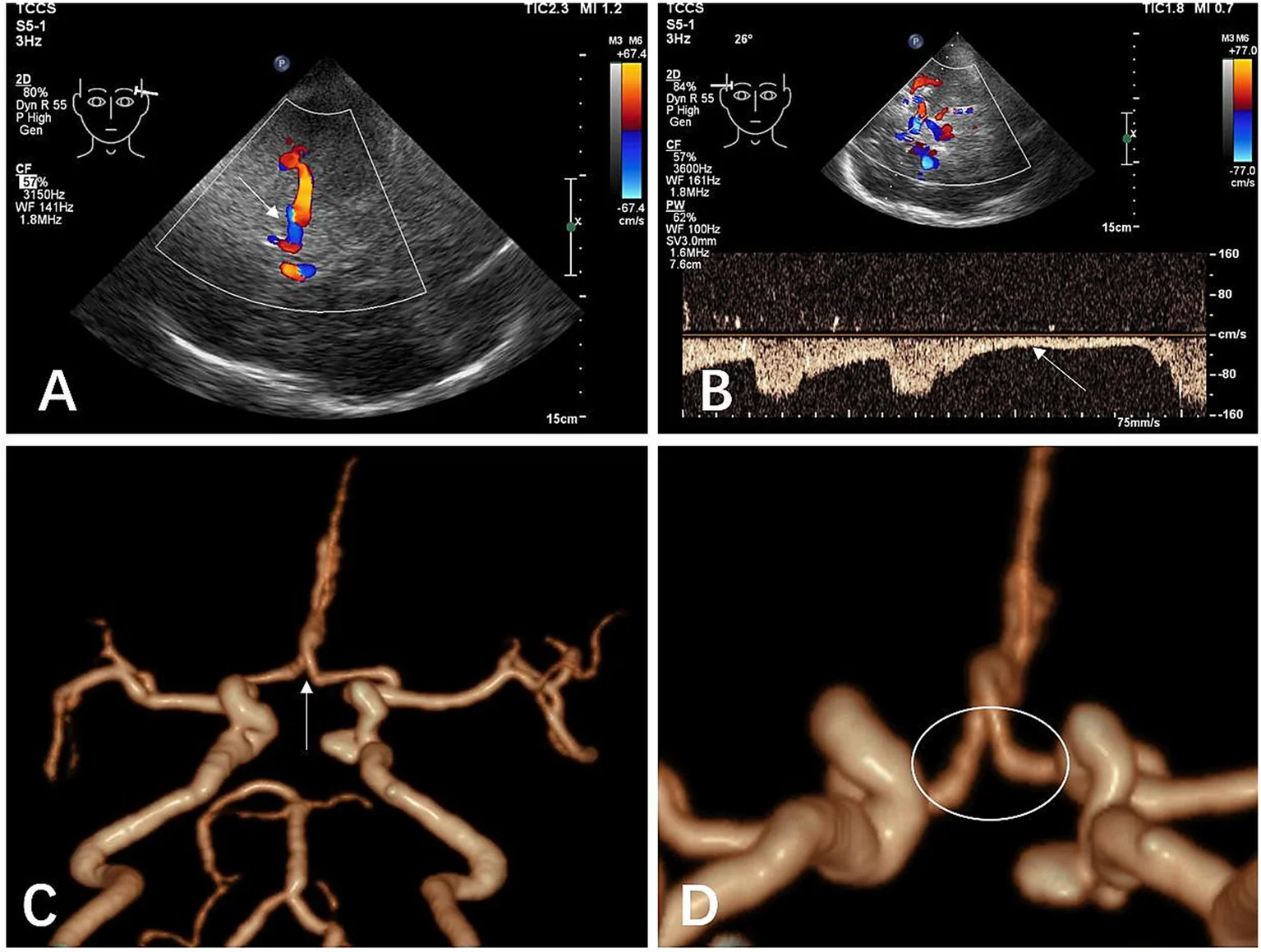
A male patient with 70%–99% stenosis at the origin of the right ICA, where the right side corresponds to the side of CEA. **(A)** The blood flow signal from the ACA-A1 segment on the contralateral side was normally displayed, directing the flow away from the probe (white arrow). **(B)** The blood flow signal from the ACA-A1 segment on the CEA side was normally displayed, with the blood flow directed away from the probe. When the CCA was compressed on the CEA side, the blood flow velocity in the ipsilateral ACA-A1 segment markedly decreased, nearly returning to baseline levels, with no evidence of reverse compensatory blood flow signals (white arrow). **(C,D)** CTA confirmed that the ACoA was absent.

**Figure 8:**
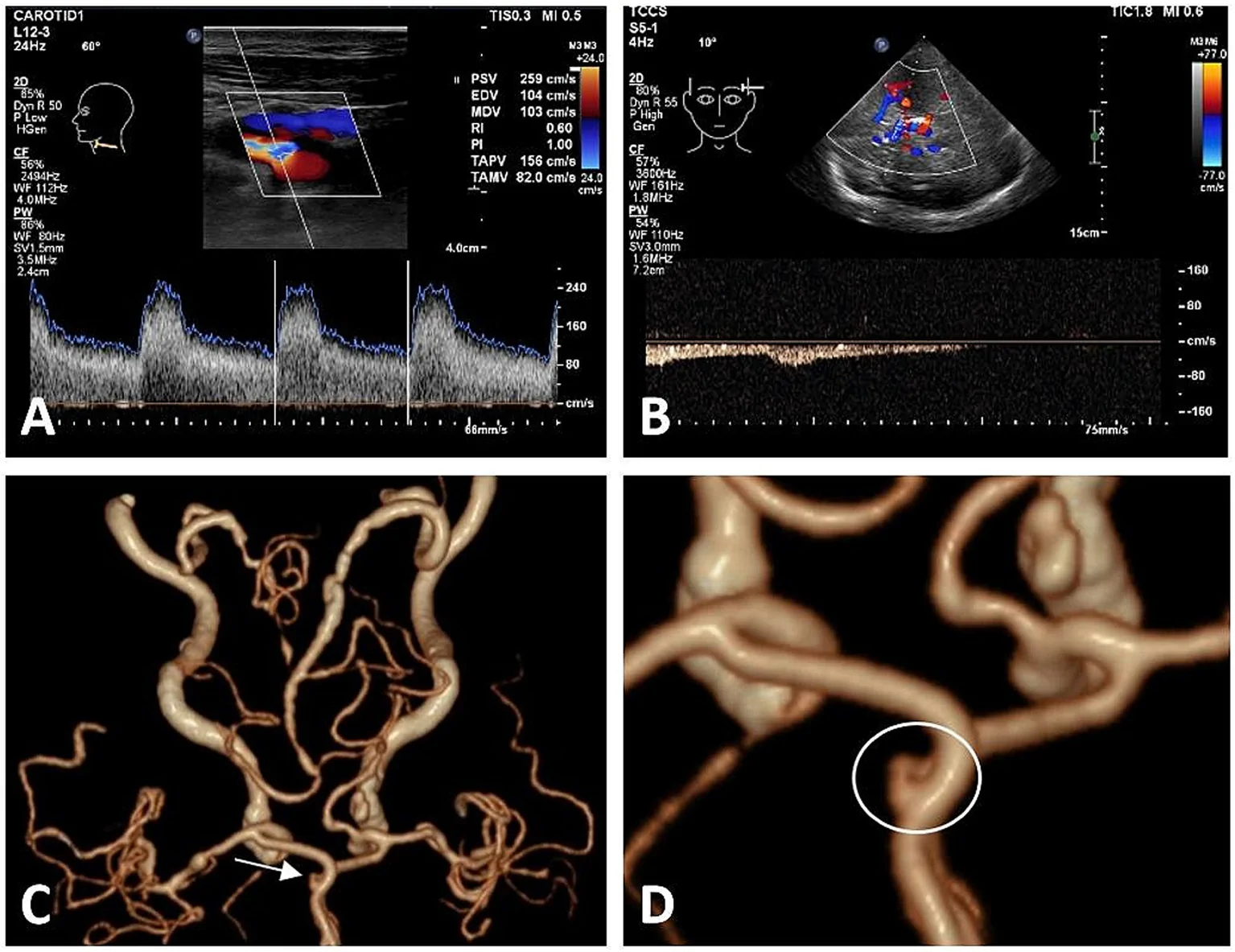
A 70-year-old male presented with numbness in the right limb. **(A)** carotid ultrasound revealed 70%–99% stenosis at the origin of the left ICA, and the left side corresponded to the side of the CEA. **(B)** CE-TCCS showed that the blood flow signal from the bilateral ACA-A1 segments was normally displayed at rest. When the CCA was compressed on the left side, the blood flow velocity in the ipsilateral ACA-A1 segment decreased significantly to baseline levels. CE-TCCS combined with CCC test demonstrated that the ACoA was not patent. **(C,D)** CTA showed that the ACoA was patent.

CE-TCCS combined with the CCC test detected functional non-patency of the ACoAs in 17 cases, consistent with CTA findings (Figure 7). However, discrepancies in the diagnostic results were observed in 4 cases between CTA and CE-TCCS combined with the CCC test. Specifically, the ACoA was anatomically patent on CTA but functionally non-patent according to CE-TCCS plus the CCC test (Figure 8).

#### 3.2.2 CE-TCCS and CTA evaluating ACoA patency

CE-TCCS identified 21 functional patent ACoAs (Figure 5A) and 71 functional non-patent ACoAs. Comparison with CTA results revealed a statistically significant discrepancy (*p* < 0.001) and poor consistency (Kappa = 0.126, 95% CI: 0.053–0.199, *p* = 0.013). CE-TCCS showed a patency rate of 22.8% (21/92) for the ACoA. 70.4% (50/71) of functional patent ACoAs in patients with normal flow in ACA-A1 segments on the CEA side were missed by CE-TCCS alone.

## 4 Discussion

Ultrasound beams undergo marked attenuation when passing through the skull. Consequently, intracranial structures cannot be clearly visualized in many elderly individuals, particularly older women and patients with inadequate temporal bone acoustic windows (24). The new-generation lung-metabolizable ultrasound contrast agent SonoVue consists of microbubbles with varying sizes, enabling it to cover the full range of frequencies applied in ultrasound imaging. Accordingly, robust contrast-specific signals can be acquired using all suitable probes across low to high frequencies (25). Furthermore, CE-TCCS achieves improved visualization of nearly all central and peripheral segments of intracranial arteries. Our study demonstrated that CEUS significantly improved the detection rate of intracranial vessels in patients with poor temporal bone windows, increasing the successful visualization rate from 32% to 65% with conventional TCCS to 89%–100% with CE-TCCS. These findings are largely consistent with the results reported by Liu et al. (18), who confirmed that CE-TCCS enables valid diagnostic insonation in 94%–100% of cases.

However, the AcoA has a mean length of 2.6 mm, but it can be as short as 0.3 mm (26). In such cases, the proximal parts of the ACAs are close to each other. These factors combine to render detection of the ACoA difficult with conventional TCCS and CE-TCCS, especially when blood flow directions in the ACoA and ACA are identical (27). Visualization of the ACoA on conventional MRA is often limited by inadequate spatial resolution (21). Currently, digital subtraction angiography (DSA) serves as the gold standard for assessing intracranial collateral circulation anatomy (28). Given the various complications associated with DSA, including arterial dissection, neurological deficits, and puncture-site hematoma, CTA represents an alternative noninvasive imaging modality (29, 30). Compared with DSA, CTA exhibits high diagnostic efficacy for evaluating the anatomical patency of the ACoA (29). In addition, CTA carries risks of radiation exposure, contrast-induced nephrotoxicity and hypersensitivity reactions (23, 29). Ultrasound contrast agents are highly safe, with adverse events primarily hypersensitivity or vasovagal reactions occurring in fewer than 0.01% of administrations (17). CE-TCCS allows real-time imaging with merits including convenience, cost-effectiveness, no ionizing radiation, lower allergic risk, and safety for patients with renal insufficiency (25). Rapid microbubble washout permits repeated injections for blood flow assessment (16). Combined with the CCC test to simulate intraoperative CCA clamping during CEA, CE-TCCS can accurately determine the functional patency of the ACoA by analyzing flow dynamics in the ACA-A1 segment.

In our study, standalone CE-TCCS showed a functional patency rate of only 22.8% (21/92) for the ACoA. However, A previous study on ICA-selective MRA reported that 73.4% of patients with an average ICA stenosis rate exceeding 70% had patent ACoAs and underwent CEA. In these cases, bilateral ACA-A2 segments received blood supply from the contralateral ICAs, whereas the ACA-A1 segments on the stenotic side were supplied by the ipsilateral ICAs with normal flow direction (8). Therefore, standalone CE-TCCS may lead to a significant missed diagnosis of a nonrecruited but functional patent ACoA when the blood flow direction in the ACA -A1 segment on the CEA side appears normal.

Our study showed that the functional patency rate of the ACoA assessed by CE-TCCS combination with the CCC test was 77.2% (71/92), which is consistent with previous report (31). We found that the combination of CE-TCCS and the CCC test detected 70.4% (50/71) of functionally patent ACoAs with normal blood flow in the CEA-side ACA-A1 segment, where flow reversal could be triggered during CCA compression. The CCC test can contribute to a pressure difference that reaches a certain threshold level within the bilateral ICA system, after which blood is abruptly diverted to available conduits, following the path of least resistance. This combined protocol is especially valuable for detecting functionally patent ACoAs that present with unaltered antegrade flow in the ipsilateral ACA-A1 segment.

CE-TCCS combined with CCC test showed good diagnostic performance and strong agreement with CTA for the assessment of ACoA patency in our study. However, discrepancies were observed in four cases. CTA identified the ACoA as anatomically patent, whereas CE-TCCS combined with the CCC test indicated functional non-patency. This suggests that while the functional patency of the ACoA corresponds to its anatomical patency, the presence of anatomical patency does not necessarily guarantee functional patency. Previous research has indicated that functional assessment is more accurate than morphological assessment alone in evaluating collateral flow (32). There are several potential explanations: first, disorganized vascular smooth muscle cells and intimal thickening may lead to structural abnormalities within the intraluminal environment of the ACoA (33); second, vascular spasm may result in the absence of blood flow through the ACoA during the CCC test; additionally, the low angle between the ACoA and ACA-A1 increases blood flow resistance and may reduce the collateral compensation efficiency of the ACoA (34).

There were some limitations to the current study. First, although the CCC test plays an important role in assessing collateral circulation via the CoW, the technique has several shortcomings, including patient discomfort, operator dependence, and potential safety risks such as bradycardia, hypotension, transient neurological deficits, plaque embolization, and visual disturbance (35). No adverse events were observed in our study. Second, this study is that CTA rather than DSA served as the reference standard. CTA only demonstrates anatomical ACoA patency but cannot reflect its functional collateral compensation. Disparate evaluation endpoints between the two imaging modalities introduced bias that may skew the diagnostic efficacy and concordance values of the CE-TCCS combined with CCC test, and conflicting imaging findings lacked verification via an objective gold standard. Third, the collapse rate of the CCA cannot be quantitatively assessed during the carotid compression test. To evaluate the effectiveness of carotid compression, we monitored blood flow in the supratrochlear artery; complete artificial occlusion of the CCA resulted in reduced or absent flow, or a reversal of flow direction in this vesse (36). Fourth, there are numerous methods for monitoring brain perfusion during clamping, including electroencephalography, stump pressure, TCD, transcranial cerebral oximetry, etc., but CE-TCCS combined with the CCC test provides a preoperative evaluation of single collateral circulation for CEA. Finally, single-center recruitment and rigid inclusion criteria led to potential selection bias. Future multicenter prospective studies with consecutive patient enrollment should be conducted. We will further explore the compensatory capacity of the ACoA in terms of quantitative measurements, and more attention should be paid to the novel TCCS grading system for collateral circulation (37).

## 5 Conclusion

Collateral arterial pathways and their clinical evaluation represent highly complex issues. For patients with insufficient temporal bone windows, CE-TCCS significantly enhanced the visualization of the CoW. CE-TCCS combined with the CCC test, as an extension of previous work, was particularly useful for identifying functionally patent ACoAs with normal blood flow in the ACA-A1 segment on the CEA side; these ACoAs would be missed by CE-TCCS alone. Although our study revealed high consistency between functional ACoA patency evaluated via CE-TCCS combined with CCC test and anatomical patency defined on CTA, discrepancies were observed in several individual cases. This suggests that anatomical patency of the ACoA does not guarantee functional patency. Therefore, CE-TCCS combined with the CCC test can serve as a reliable complement to conventional imaging modalities for preoperative evaluation of ACoA patency. This technique facilitates the formulation of neuroprotective strategies, thereby improving patient safety and strengthens surgeons’ confidence in preoperative planning.

## Data Availability

The original contributions presented in the study are included in the article/supplementary material, further inquiries can be directed to the corresponding authors.
